# Membrane-Associated RING-CH Proteins Associate with Bap31 and Target CD81 and CD44 to Lysosomes

**DOI:** 10.1371/journal.pone.0015132

**Published:** 2010-12-02

**Authors:** Eric Bartee, Craig A. Eyster, Kasinath Viswanathan, Mandana Mansouri, Julie G. Donaldson, Klaus Früh

**Affiliations:** 1 Vaccine and Gene Therapy Institute, Oregon Health and Science University, Beaverton, Oregon, United States of America; 2 Department of Molecular Genetics and Microbiology, College of Medicine, University of Florida, Gainesville, Florida, United States of America; 3 Laboratory of Cell Biology, National Heart, Lung, and Blood Institute (NHLBI), National Institutes of Health (NIH), Bethesda, Maryland, United States of America; Yale Medical School, United States of America

## Abstract

Membrane-associated RING-CH (MARCH) proteins represent a family of transmembrane ubiquitin ligases modulating intracellular trafficking and turnover of transmembrane protein targets. While homologous proteins encoded by gamma-2 herpesviruses and leporipoxviruses have been studied extensively, limited information is available regarding the physiological targets of cellular MARCH proteins. To identify host cell proteins targeted by the human MARCH-VIII ubiquitin ligase we used stable isotope labeling of amino-acids in cell culture (SILAC) to monitor MARCH-dependent changes in the membrane proteomes of human fibroblasts. Unexpectedly, we observed that MARCH-VIII reduced the surface expression of Bap31, a chaperone that predominantly resides in the endoplasmic reticulum (ER). We demonstrate that Bap31 associates with the transmembrane domains of several MARCH proteins and controls intracellular transport of MARCH proteins. In addition, we observed that MARCH-VIII reduced the surface expression of the hyaluronic acid-receptor CD44 and both MARCH-VIII and MARCH-IV sequestered the tetraspanin CD81 in endo-lysosomal vesicles. Moreover, gene knockdown of MARCH-IV increased surface levels of endogenous CD81 suggesting a constitutive involvement of this family of ubiquitin ligases in the turnover of tetraspanins. Our data thus suggest a role of MARCH-VIII and MARCH-IV in the regulated turnover of CD81 and CD44, two ubiquitously expressed, multifunctional proteins.

## Introduction

Ubiquitination plays a key role in regulating many diverse cellular functions, predominantly by tagging proteins for destruction via either the proteasome or lysosome. The ubiquitin pathway consists of ubiquitin itself, a single ubiquitin activating enzyme (E1), a small number of ubiquitin conjugating enzymes (E2's), and a wide variety of ubiquitin ligase enzymes (E3's). Due to the limited variability in E1 and E2 enzymes, much of the regulation of the ubiquitin pathway is carried out by the E3's [1]. E3 enzymes provide specificity to the ubiquitin pathway by linking E2 enzymes with their substrates.

Membrane associated RING-CH (MARCH) proteins, belong to a family of transmembrane ubiquitin ligases (for a recent review see: [2]) that was initially discovered when RING-CH proteins encoded by gamma-2 herpesviruses (KSHV-K3, KSHV-K5, MHV68-K3) were shown to down-regulate the surface expression of transmembrane immune-stimulatory host cell proteins, particularly MHC class I, thus contributing to viral immune evasion [3], [4], [5]. Leporipoxviruses encode similar MHC-I down-regulating proteins which contribute to viral virulence [6], [7]. This immunoreceptor down-regulation is accomplished by the viral proteins ubiquitinating lysines in the cytoplasmic tails of their transmembrane substrates [8]. In the absence of lysines, tyrosines, serines and threonines can also be ubiquitinated [9], [10]. Essential for ubiquitination is the RING-CH domain which is structurally similar to canonical RING-HC and RING-H2 domains [11]. Depending on the intracellular site of ubiquitination the same target proteins can be either degraded by the proteasome via ER-associated degradation or ubiquitin-mediated targeting to multivesicular bodies (MVB) followed by lysosomal degradation [12]. The sequence and structural homology of the viral MARCH proteins to host MARCH family suggested that the viral proteins were pirated from ancestral host proteins that likely perform related functions.

Vertebrate MARCH family members fall into distinct classes according to their sequence relatedness and number of transmembrane domains [13]. Structurally most similar to the viral MARCH proteins, are the two-transmembrane spanning proteins MARCH-I, -II, -III, -IV, VIII, IX and XI [14], [15]. We and others previously demonstrated that several known target proteins of viral MARCH homologues can also be targeted by human MARCH proteins [13], [14], [16], [17]. Additionally, the closely related MARCH-I and MARCH-VIII were found to ubiquitinate MHC class II, a protein that is not targeted by any of the viral proteins [18], [19]. This ubiquitin-mediated MHC-II turnover via MARCH proteins seems to play a crucial role in the regulation of antigen-presentation by dendritic cells, macrophages and B-cells [20], [21], [22], [23]. However, aside from these studies, relatively few physiological cellular targets for MARCH proteins have been identified to date [2].

To identify novel targets for MARCH proteins we previously adapted a quantitative proteomics-based method termed stable isotope labeling with amino acids in cell culture (SILAC) [24], [25] and monitored KSHV K5-dependent changes in the plasma membrane proteome [14]. By comparing the relative abundance of tryptic peptides identified by mass spectroscopy in membrane fractions of KSHV-K5 expressing HeLa cells labeled with ‘heavy’ amino acids (C^13^/N^15^) prior to control cells labeled with ‘light’ (C^12^/N^14^) amino acids, we were able to identify and independently confirm several novel substrates for KSHV-K5 [14]. Most notably, this work was the first indication of viral proteins targeting activated leukocyte cell adhesion molecule (CD166) and the interferon-induced anti-viral host cell factor BST2/Tetherin [26]. More recently, SILAC was used to determine changes in the plasma membrane proteome of B cell lines stably expressing MARCH-IX [27]. Several novel targets were confirmed by flow cytometry thus validating this approach to identify cellular proteins that are targeted by MARCH family proteins.

Based on quantitative membrane proteomics identification of novel membrane-associated proteins down-regulated by MARCH-VIII in primary human fibroblasts, we now show that the ER resident chaperone Bap31 is a binding partner for the majority of MARCH proteins and that Bap31 controls their intracellular transport. In addition, we identify two novel targets for MARCH-VIII. Specifically, show that the hyaluronic acid receptor CD44 and the tetraspanin CD81 are internalized and targeted to lysosomes in the presence of MARCH-VIII. CD81 expressed in fibroblasts was additionally targeted by MARCH-IV since over expression of MARCH-IV redirected CD81 to lysosomes whereas siRNA treatment against endogenously expressed MARCH-IV increased CD81 surface levels. Thus, CD44 and CD81 likely represent physiological substrate of MARCH-I and VIII. CD44 and CD81 perform multiple functions including the regulation of cell-cell interactions within the immune system and elsewhere. Our data suggest that these proteins can be regulated by ubiquitination via MARCH proteins.

## Materials and Methods

### Reagents

The following antibodies were used: anti-CD44 (clones F-4 and DF1485) and anti-CD81 (clone 5A6) (Santacruz Biotechnology), anti-Bap31 (Affinity Bioreagents), anti-TfR (US Biologicals), anti-Flag (Sigma). Plasmids expressing MARCH cDNA's have been described previously [13]. Ad-MARCH-VIII and Ad-K5 have been described previously [6], [14]. Ad-Vpu was a generous gift from A. Moses (Vaccine and Gene Therapy Institute, OR, USA) [28].

### Cells

Human foreskin fibroblasts were obtained from ATCC and maintained in DMEM (Invitrogen) supplemented with 10% fetal calf serum (Hyclone) and 1× pen/strep.

### Stable isotope labeling of HFF cells

Cells were labeled with stable isotopes using labeling medium (DMEM, Invitrogen) lacking the amino acids l-lysine and l-leucine (prepared according to the manufacturer's protocol). Medium was supplemented with 10% dialyzed fetal calf serum (Hyclone), 1× Pen/Strep (Invitrogen), and either isotopically light l-lysine and l-leucine (Sigma) or isotopically heavy l-lysine (U-13C6, 98%; U-15N2, 98%) and l-leucine (U-13C6, 98%; 15N, 98%) (Cambridge Isotope). Cells were maintained in labeling medium long enough to allow for 6 doublings prior to initiation of the experiment to ensure complete labeling.

### Preparation of samples for MS/MS analysis

Cells grown in labeling medium were infected with either Ad-Tet + Ad-MARCH-VIII (heavy-labeled cells) or Ad-Tet (light-labeled cells) alone at an MOI of 200. 24 hrs post-infection, cells were harvested by scraping, washed twice in PBS, resuspended in PBS containing 5 mM EDTA, and lysed by douncing. Unlysed cells and debris were cleared from the lysate by centrifugation for 5 min at 3,000×*g*. The cleared lysates were separated into membrane and soluble fractions by centrifugation for 30 min at 45,000×*g*. The membrane fraction was resuspended in PBS by sonication and separated over a discontinuous sucrose gradient (2 M, 1.6 M, 1.25 M, 1.2 M, and 0.8 M) by centrifugation for 2.5 h at 25,000 rpm (Sorvall SW-28 rotor). The bands corresponding to the plasma membrane (0.8–1.2 M interphase), Golgi (1.25−1.2 M interphase), and ER (1.6–1.25 M interphase) fractions were removed, diluted 5× in Tris-EDTA (pH 8.0), and centrifuged for 30 min at 45,000×*g* to pellet the proteins contained in each fraction. Pellets were washed for 30 min in 50 mM sodium bicarbonate (pH 11.5) and centrifuged for 30 min at 45,000×*g*, followed by a second wash in 50 mM ammonium bicarbonate (pH 8.5) and further centrifugation for 30 min at 45,000×*g*. Final pellets were resuspended in 8.0 M deionized urea and 50 mM ammonium bicarbonate (pH 8.5) and protein levels quantitated using the Bio-Rad Protein Assay (Bio-Rad). Samples were reduced with DTT (Sigma) and alkylated with iodoacetamide (Sigma) prior to overnight digestion with trypsin (Promega).

### Chromatography, MS, and informatics

Peptide mixtures were analyzed by electrospray ionization tandem MS, coupled to two-dimensional liquid chromatography, which was performed using a modified version of the protocol described by Link and coworkers [29]. Briefly, 22 µg of sample was loaded onto an Opti-Pak capillary SCX trap cartridge (Optimize Technologies) and eluted stepwise (12.5, 25, 37.5, 50, 62.5, 75, 87.5, 100, 112.5, 125, 200, 300, or 450 mM ammonium acetate in 0.1% formic acid) onto a reverse phase C-18 capillary column (180 um×100 mm, BioBasic-18; Thermo Electron). Peptides were then eluted using an acetonitrile gradient (5%, 5 min; 5%–40%, 75 min; 40%–90%, 10 min) into a ProteomeX LCQ Deca XP Plus mass spectrometer (Thermo Electron) equipped with a low-flow (1 µl/min) electrospray source. The instrument was set to trigger data-dependent MS/MS acquisition of the three most intense ions detected during the MS survey scan when total ion current per MS survey scan exceeded 5.0×10^5^ counts.

Proteins were identified by analyzing tandem mass spectra with the Sequest algorithm (Thermo Electron) as described [30] using the human subset of the UniProt/Swiss-Prot protein database (UniProt release 5.1, http://www.expasy.org/sprot). The search results were further analyzed using PeptideProphet [31]. SILAC ratios were determined using the ASAPRatio algorithm [32]. Multiple peptides derived from a single protein were included if PeptideProphet probability was greater than or equal to 0.85. All positive results were manually verified.

### Real-Time PCR

Short (50–100 bp) fragments from MARCH-I, MARCH-II, MARCH-IV, MARCH-VIII and MARCH-IX cDNA were amplified via PCR using appropriate primers. The PCR reaction was carried out in the following buffer: 1× SYBR Green PCR Buffer (PE Biosystems), 3 mM MgCl_2_, 0.8 mM dNTP's, 0.625 U Amplitaq Gold (PE Biosystems), 0.01 µl Amperase (PE Biosystems), and 50 nM primers. 20 µl buffer was added to 5 µl template cDNA and run under the following conditions: 95°C for 10 min, followed by 40 cycles of 95°C for 15 seconds, 60°C for 1 minute. Amplification was tracked via SYBR-Green (PE Biosystems) incorporation using an ABI-PRISM 7700 Sequence Detection System (Applied Biosystems).

### Co-immunorecipitation, Western Blot, and Surface Labeling

Cells were washed twice with PBS and lysed in PBS containing 1% CHAPS (Sigma). Lysates were pre-cleared with protein A/G agarose beads and incubated with 1 µg of antibody for 1 hr and followed by 1 hr incubation with protein A/G beads. Immunoprecipated proteins were washed four times with PBS containing 1% CHAPS. Samples were boiled in SDS buffer and analyzed by SDS-PAGE gel electrophoresis. For Western blot analysis, SDS-PAGE gels were transferred to Immobilon-P PVDF membrane (Millipore) using semidry transfer. Following transfer, membranes were blocked in 10% milk in PBS +1% tween-20. Membranes were incubated with primary and secondary antibodies in 5% milk for 30 minutes. Following each antibody incubation, membranes were washed 3 times with PBS +1% tween-20, and once with dH_2_O.

Surface biotinylation studies were carried out using the Cell Surface Protein Biotinylation and Purificaiton Kit (Pierce) according to the manufacturer's recommendations.

### Flow Cytometry

Cells were removed from tissue culture dishes with 0.05% trypsin-EDTA (Invitrogen), washed with ice-cold PBS, and incubated with appropriate antibody for 30 min at 4°C. The cells were washed with ice-cold PBS and either resuspended in ice-cold PBS or incubated with PE-conjugated anti-mouse secondary antibody (Dako) and washed again before analysis with a BD Biosciences FACSCalibur flow cytometer.

### Immunofluorescence

HeLa cells were plated on glass cover slips and 24 h later were transfected with MARCH-FLAG constructs using FuGene according to manufacturer's specifications (Roche). After 24 h of transfection, cells were fixed for 10 min in 2% formaldehyde in PBS, rinsed twice in PBS, and block with 10% FBS/PBS for 20 min. Cells were incubated with primary antibodies in 10% FBS/PBS in the presence of 0.2% saponin for 1 h. Cells were washed 3× with 10% FBS/PBS and incubated with Alexa-dye conjugated secondary antibodies for 1 h to detect primary localizations. Cells were washed twice with 10% FBS/PBS, twice with PBS, and mounted on slides using Fluoromount-G (SouthernBiotech). All images were obtained using a 510 LSM confocal microscope (Zeiss) with 63× Plan Apo objective. Adobe photoshop was used for image processing.

### siRNA Treatment

Knockdown of Bap31 was accomplished using anti-BCAP-31 (Bap31) siGENOME siRNA (Dharmacon). siRNA transfection was accomplished using Oligofectamine (Invitrogen) according to manufacturers recommendations. 25% confluent 35 mm dishes of HFF's were treated twice with 5 µl siRNA (20 µM). Treatments were 6 h apart. Three days post treatment, cells were treated again with siRNA as described. Cells were analyzed 4 days after the second siRNA treatment.

## Results

### Proteomic Analysis of MARCH-VIII-expressing human fibroblasts

To identify novel substrates for MARCH-VIII proteins in human cells, we used primary human foreskin fibroblasts (HFF). These cells were selected for several reasons: 1) HFFs express low levels of most MARCH proteins, thus allowing validation of identified targets by siRNA treatment (see below); 2) they are primary human cells; 3) they grow robustly allowing the five doublings required for complete incorporation of the stable isotopes [24], [25]. To detect changes in the membrane proteomes of HFFs upon forced-expression of MARCH-VIII we compared the abundance of proteins that were metabolically labeled using stable isotopes and identified by tandem MS/MS. HFFs were grown in either ‘light’ media containing normal lysine and leucine, or ‘heavy’ media containing N^15^/C^13^-labeled leucine and lysine. To ensure complete labeling, cells were grown in labeling media for six doublings (∼2 weeks). Following metabolic labeling, ‘light’ labeled samples were infected with Adenovirus expressing the Tetracycline regulatable-transactivator (Ad-Tet) alone, while ‘heavy’ labeled’ samples were infected with Ad-Tet together with Tet-induced Ad-MARCH-VIII. 24 h post infection, cells were harvested via mechanical scraping and cellular fractions were separated as described [14] except that only the plasma membrane (PM) fraction was analyzed. Following trypsinization, PM samples were separated with two-dimensional liquid chromatography (strong cation exchange followed by reverse phase chromatography) and then analyzed by mass-spectrometry using a LCQ ion-trap instrument in data-dependent MS/MS mode. Ion spectra were identified using SEQUEST software and statistically analyzed using PeptideProphet. To limit false positive peptide identifications a PeptideProphet cutoff of 0.85 was applied. To further minimize experimental error and limit false positive results, three biological replica experiments were prepared and independently analyzed. Each experiment resulted in the identification of between 300–500 unique proteins (data not shown).

To determine proteins whose expression was altered by MARCH-VIII, ratios between heavy and light peptide peaks were determined using the ASAPRatio algorithm [32]. Ratios of individual peptides belonging to the same protein were grouped together using ProteinProphet to obtain a ratio for the expression of each identified protein [32]. HFFs express three known substrates of MARCH-VIII: Transferrin receptor (TfR), MHC class I, and activated leukocyte adhesion molecule (ALCAM, CD166) [13], [14]. However, we failed to recover peptides corresponding to TfR or the MHC I heavy chain in any of the experiments. This is likely due to the lower expression levels of both TfR and MHC I in HFFs compared to HeLa cells previously used. In contrast, the light chain of the MHC I heterodimer, β2-microglobulin (β2m), was recovered in all three experiments (**Table 1**). The recovery of β2m but not MHC I heavy chain might be due to the fact that β2m is non-polymorphic in contrast to the multiple MHC I alleles present in the mixed population of primary fibroblasts. Since peptides derived from the known MARCH-VIII substrate ALCAM were recovered in only two of the three experiments, we also considered proteins identified in two out of three experiments as likely targets. Thus, proteins displaying a ratio in the top or bottom 25% of two of the three experiments were considered potential candidates if they were absent from the third experiment due to a failure to recover any peptides. Using this initial filter resulted in the identification of 13 candidate protein targets for MARCH-VIII (**Table 1**).

**Table 1:** Proteins displaying a differential abundance following MARCH-VIII expression.

Protein	Average Ratio	Identified in	Unique Peptide
**Downregulated**			
B2MG	2.41	3/3	2
CD44	1.70	3/3	6
CD81	3.10	3/3	2
Bap31	2.35	2/3	1
CD166	2.93	2/3	4
CD9	2.43	2/3	3
DAG1	2.81	2/3	1
ECHA	2.51	2/3	1
EGLN	2.29	2/3	2
RALA	3.06	2/3	1
RL15	3.02	2/3	1
**Upregulated**			
MARCH-VIII	0.10	3/3	3
Grp78	0.85	3/3	11

13 proteins displayed a change in abundance of more than 2 fold following transduction with Ad-MARCH-VIII in two of three replicate experiments. Listed are the Uniprot designations for each protein, as well as the average ratio observed. Ratios given are Ad-Tet:Ad-MARCH-VIII. Ratios>1 correspond to proteins that are downregulated by expression of MARCH-VIII while ratios <1 correspond to proteins that are upregulated by expression of MARCH-VIII. Also given are the number of unique peptides used to identify each protein and whether that protein was changed in 2/3 or 3/3 experiments.

Of these 13 proteins, only two proteins displayed increased abundance upon expression of MARCH-VIII: MARCH-VIII itself and Grp78/Bip. Grp78/Bip is an ER resident chaperone that is transcriptionally up-regulated as part of the unfolded protein response, which is triggered by elevated levels of viral or cellular proteins in the ER. Therefore, it is likely that up-regulation of Grp78 is due to the forced expression of MARCH-VIII. However, this has yet to be confirmed experimentally.

All remaining 11 proteins displayed lower levels in Ad-MARCH-VIII-transduced cells compared to control cells. Of these proteins, three were down-regulated in all three experiments: β_2_M, CD44, and CD81. Since MHC-I is known to be moderately down-regulated by MARCH-VIII [13], it is likely that the observed reduction of β2m, is caused by the removal of MHC-I from the cell surface. Alternatively, it is conceivable that MARCH-VIII also down-regulates some of the non-classical HLA-like molecules that associate with β_2_m. However, we did not recover non-classical MHC molecules in any of our experiments. Since expression levels for both CD44 and CD81 were consistently lower in the presence of MARCH-VIII in all three experiments, these molecules represented the most likely candidates for novel MARCH-VIII substrates. CD44 is a type I transmembrane glycoprotein that connects a variety of extracellular matrix proteins, most notably hyaluronic acid (HA), to the cell surface [33]. CD81 is a member of the tetraspanin family which together form the ‘tetraspanin-web’ that regulates the lateral localization of other surface proteins [34].

Proteins down-regulated in two of three experiments were considered less likely to be true substrates for MARCH-VIII. In fact, most of the proteins found in this class seemed unlikely targets for MARCH-VIII due to their predicted subcellular localization in contaminating fractions of our membrane preparation such as cytosolic, nuclear or mitochondria (data not shown). However two proteins, CD9 and Bap31, represented potentially true positives due to their transmembrane structure and location at the plasma membrane or intracellular vesicular compartments. Like CD81, CD9 is a surface glycoprotein of the tetraspannin family. CD9 and CD81 interact with each other and regulate many of the same cellular processes [35]. Bap31 is a molecular chaperone that regulates intracellular trafficking of a number of cell surface proteins, including tetraspanins as well as MHC-I [36], [37], [38], [39]. Therefore, we included CD9 and Bap31 for further validation studies.

### Bap31, but not CD9 is removed from the cell surface by MARCH-VIII

To validate the proteomics results, we examined CD9 expression by flow-cytometry in both HeLa cells and HFFs. However, expression of MARCH-VIII in either cell type did not result in any down-regulation of CD9 from the cell surface (data not shown). Thus, the lower levels of CD9-derived peptides seemed to be a false-positive result caused by inter-experimental variation.

**Figure 1:**
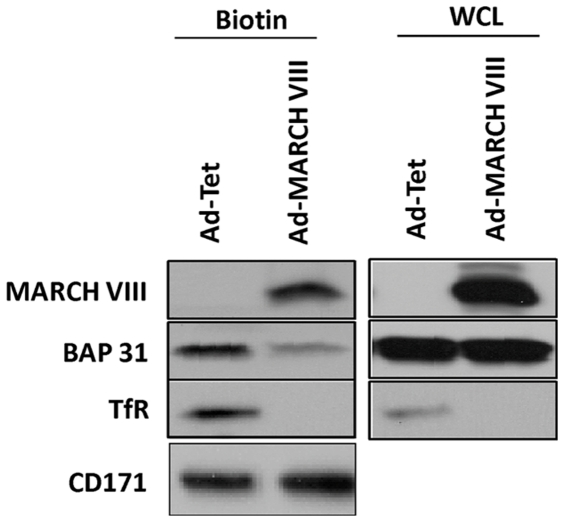
MARCH-VIII reduces surface expression of Bap31. HFF's were transduced with Ad-Tet and Ad-MARCH-VIII or Ad-Tet alone. 24 hours post-infection, cells were biotinylated. Prior to purification of biotinylated proteins, 100 µl of each sample was removed for analysis of protein expression in each whole cell lysate (WCL). Expression of Bap31 was analyzed in both the biotinylated fraction and the whole cell lysate using immunoblot. Expression of MARCH-VIII and the known MARCH-VIII substrate transferrin receptor (TfR) were included as controls.

Unlike ALCAM and CD9, Bap31 is an ER resident chaperone associated with the forward transport of a wide variety of cell surface proteins. In addition to MHCI, Bap31was shown to regulate the intracellular of p450 [40], gamma actin [41], cellubrevin [42], cystic fibrosis transmembrane conductance regulator [43], membrane bound IgD [44], CD18 and CD11b [45] as well as HPV E5 [46]. In addition, Bap31 has been implicated in retro-translocation during ER-associated protein degradation [47]. To determine whether MARCH-VIII reduces Bap31 levels, we initially measured total Bap31 levels in Ad-MARCH-VIII transduced HFFs by immunoblot. However, total Bap31 levels were not changed (**Fig. 1**).

While most Bap31 is in the ER, a small portion of Bap31 is present in both the Golgi apparatus and at the plasma membrane [45], [48]. Since our initial proteomic screen showed a reduction of Bap31 levels in a fraction highly enriched for the plasma membrane, we hypothesized that expression of MARCH-VIII might affect the small portion of Bap31 which localizes to the cell surface. To test this hypothesis, HFFs were infected with Ad-Tet or Ad-MARCH-VIII and, 24 hours post infection, proteins at the cell surface were labeled with Biotin using the Cell Surface Biotinylation and Purification Kit (Pierce). The biotinylated fraction was then purified according to the manufacturer's recommendations. A small portion of each whole cell lysate (WCL) was removed prior to biotin purification to determine total Bap31 levels in the lysate. Both biotinylated and WCL fractions were separated using SDS-PAGE and the levels of MARCH-VIII, Bap31, and TfR were analyzed by immunoblot (**Fig. 1**). While the majority of MARCH-VIII was seen in the WCL fraction, MARCH-VIII was also present in the biotin fraction. This suggests that a small portion of MARCH-VIII traffics to the cell surface. As previously described, a small portion of Bap31 was seen in the biotin fraction, suggesting a cell surface localization [45]. Interestingly, the levels of biotinylated Bap31 were significantly reduced following expression of MARCH-VIII. In contrast, total levels of Bap31 in the WCL fraction remained unchanged whereas expression of MARCH-VIII strongly reduced levels of TfR in both the biotinylated and WCL fractions consistent with previously reported flow data [13]. These data suggested that MARCH-VIII does not degrade Bap31 localized to the ER, but either inhibits Bap31 trafficking to the cell surface or degrades surface expressed Bap31.

**Figure 2:**
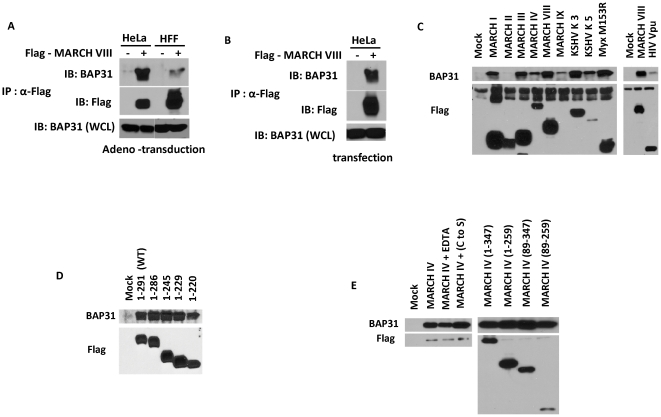
MARCH-VIII interacts with Bap31 through the transmembrane domains. MARCH-VIII C-Flag was expressed in HeLa cells and HFFs using either transfection (A) or adenoviral transduction (B). Cells were lysed in 1% CHAPS and MARCH-VIII was immunoprecipitated using the anti-Flag antibody. Following immunoprecipitation, samples were separated on an SDS-PAGE gel and the presence of Bap31 was analyzed via immunoblot. 5% of each sample was blotted with anti-Flag antibody to confirm MARCH-VIII expression. (C): HeLa cells were transfected with C-terminally Flag tagged versions of each MARCH protein as well as the viral MARCH proteins KSHV-K3, KSHV-K5, MYXV-M153 and the HIV-1 protein Vpu. 24 hours post transfection, each sample was lysed and analyzed as above. Expression of each transfected protein was confirmed by blotting 5% of each immunoprecipitation with the anti-Flag antibody. (D): To determine which region of MARCH proteins was required for interaction with Bap31 we used a series of previously constructed MARCH -VIII truncations. HeLa cells were transfected with C-terminally flag-tagged versions of truncated MARCH-VIII. 24 hours post transfection, samples were lysed and analyzed as above. (E): Similar experiments were performed with truncated MARCH-IV (right panel). Bap31 failed to coimmunoprecipitate following the removal of the entire C-terminus of MARCH-VIII (1–220) or MARCH-IV (1–259), removal of the entire N-terminus of MARCH-IV (89–347) or disruption of the MARCH-IV RING-CH domain by either mutation (MARCH-IV C to S) or addition of EDTA.

Since Bap31 interacts with a wide variety of proteins it was possible that the reduction of Bap31 surface levels was due to MARCH-VIII binding to Bap31 in intracellular membrane compartments. To examine this hypothesis we transfected a MARCH-VIII expression plasmid into both HeLa cells and HFFs. 24 hours post transfection, cells were lysed in the mild detergent CHAPS to preserve protein-protein interactions. MARCH-VIII was immuno-precipitated using an anti-Flag antibody and samples were separated using SDS-PAGE. Following transfer to PVDF membrane, the presence of Bap31 in each sample was analyzed by western blot. A strong signal for Bap31 was observed following MARCH-VIII immuno-precipitation in HeLa cells transfected with MARCH-VIII (**Fig. 2A**). This suggested that Bap31 interacted directly with transfected MARCH-VIII. 5% of each sample was blotted using anti-Flag antibody to confirm expression of MARCH-VIII. The failure to observe Bap31 in samples from transfected HFFs is likely due to low transfection efficiency since MARCH-VIII was not detected either (data not shown). In contrast, when MARCH-VIII was expressed by adenovirus transduction, Bap31 was also clearly present following MARCH-VIII immune-precipitation from HFF (**Fig. 2B**). This strongly suggests that MARCH-VIII interacts directly with Bap31 independent of the cell type.

Since Bap31 interacted with MARCH-VIII, we tested whether Bap31 would interact with other cellular or viral MARCH-proteins. Expression plasmids for each construct were transfected into HeLa cells and then analyzed as above (**Fig. 2C**). Bap31 was observed in samples transfected with virtually all MARCH proteins with the notable exception of MARCH-II despite clearly detectable MARCH-II expression. Similarly, very little Bap31 was observed in samples transfected with Vpu, a viral protein interacting with cellular ubiquitin ligases not related to MARCH proteins [28]. K5 and MARCH-IX form SDS-stable high molecular weight complexes so that very little protein corresponding to the predicted molecular weight is recovered (see also **Fig. 2E**). Nevertheless, Bap31 was recovered with each of these proteins. We conclude that Bap31 interacts with most members of the MARCH-family.

Previous interactions with Bap31 have been shown to be mediated through the transmembrane domains [39], [45]. To determine if this was true for Bap31's interactions with the MARCH proteins we used a series of N- and C-terminal truncations. Expression plasmids for each truncation were transfected into HeLa cells. Following immune-precipitation of each truncation using an anti-Flag antibody, each sample was analyzed for the presence of Bap31 by immunoblot. Deletion of the entire C-terminus of either MARCH-VIII (MARCH-VIII 1–220) or MARCH-IV (MARCH-IV 1–259) had no effect on Bap31 binding (**Fig 2D, E**). These data suggest that the interaction between Bap31 and MARCH proteins is not mediated through the C-terminus. Similarly, deletion of the entire N-terminus of MARCH-IV (MARCH-IV 89–347) or disruption of the RING-CH domain through mutational analysis (MARCH-IV C to S) or addition of the metal chelator EDTA (MARCH-IV + EDTA) did not prevent the interaction with Bap31. These results suggested that the N-terminus and RING-CH domain of MARCH proteins do not mediate the interaction with Bap31. Finally, Bap31 was also recovered following expression of a construct encoding the region 89–259 that comprises the zing finger and trans-membrane domains of MARCH-IV (MARCH-IV 89–259). Despite significantly different levels of expression, similar levels of Bap31 were recovered from cells expressing just the transmembrane domains of MARCH-IV and from wild-type MARCH-IV. This strongly argues that Bap31 interacts with the MARCH proteins through their transmembrane domains.

### Bap31 targets MARCH-VIII to the cell surface

**Figure 3:**
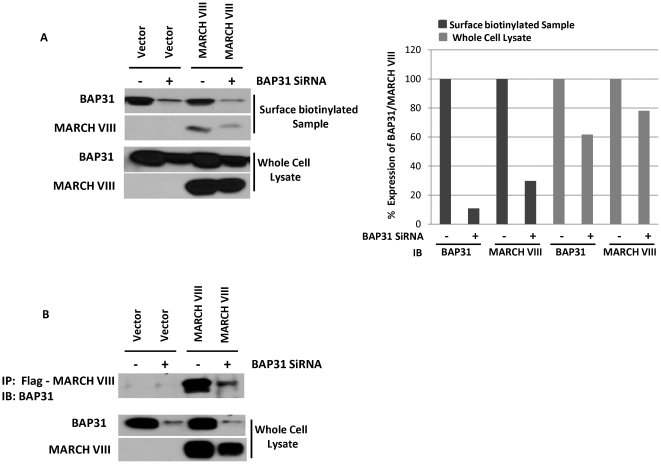
Depletion of Bap31 reduces surface expression of MARCH-VIII. HeLa cells were depleted of Bap31 using siRNA as described in the materials and methods section. (A): Following Bap31 depletion, HeLa cells were transfected with a plasmid expressing MARCH-VIII. 24 hours post transfection cells were biotinylated. Following separation via SDS-PAGE gels, surface expression (Top) or total expression (Bottom) of both Bap31 and MARCH-VIII were analyzed via immunoblot. Cells depleted of Bap31 show reduced surface expression of both Bap31 and MARCH-VIII. (B): Depletion of Bap31 also reduced the levels of Bap31 associated with MARCH-VIII. Following Bap31 depletion, HeLa cells were transfected with a plasmid expressing MARCH-VIII. 24 hours post transfection cells were lysed in 1% CHAPS and MARCH-VIII was immunoprecipitated using an anti-Flag antibody. Samples were separated via SDS-PAGE and the presence of Bap31 determined by immunoblot (B Top). Expression of Bap31 and MARCH-VIII were confirmed in whole cell lysates (B Bottom).

Since Bap31 serves as a forward cargo transporter, it was possible that MARCH proteins require this chaperone for correct folding and ER exit. To test this hypothesis we depleted HeLa cells of Bap31 using siRNA followed by transfection with MARCH-VIII. 24 hours post-transfection, the cell surface was labeled with biotin and both biotin and WCL fractions were prepared as above. Following separation using SDS-PAGE, the levels of MARCH-VIII and Bap31 in each fraction was analyzed via immunoblot (**Fig. 3A**). Although only a partial knockdown of Bap31 was achieved (**Fig. 3A, B**), the levels of Bap31 present in the biotinylated fraction were significantly reduced (**Fig. 3A** **Top**). In cells depleted of Bap31, a significantly reduced amount of MARCH-VIII was observed in the biotinylated fraction. This was not due to lower overall expression, since similar levels of MARCH-VIII were observed in the WCL fraction. Surface expression of B7.2, which is not known to interact with Bap31 was unaffected (data not shown). Despite knockdown of Bap31, some MARCH-VIII was still present in the biotinylated fraction. This might be due to incompletely knockdown of Bap31, since even in depleted cells Bap31 was still co-immuno-precipitated with MARCH-VIII (**Fig 3B**). Presumably due to the remaining MARCH-VIII, we did not observe an effect of Bap31 knockdown on the down-regulation of MARCH-VIII target proteins (data not shown). Taken together these data suggest that Bap31 supports the intracellular transport of MARCH proteins by intra-membrane interactions. Due to this interaction, Bap31 remains intracellular upon forced expression of MARCH proteins which explains its reduced presence in the plasma-membrane fraction of MARCH-VIII transduced cells. We conclude that Bap31 plays a role in the folding, assembly and intracellular transport of MARCH proteins.

### Confirmation of CD44 and CD81 downregulation

**Figure 4:**
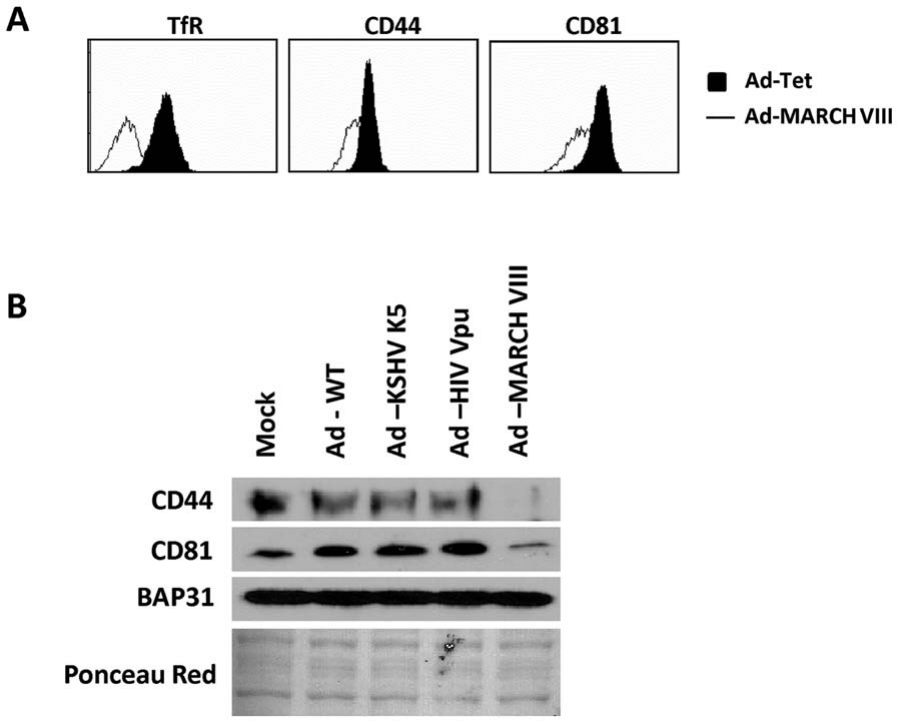
CD44 and CD81 are differentially expressed following MARCH-VIII expression. (A): To confirm that CD44 and CD81 were removed from the cell surface, HFF's were infected as above. 24 hours post-infection cells were harvested and processed for flow cytometry. Samples transduced with Ad-MARCH-VIII (line) display reduced surface expression of CD44 and CD81 compared to samples infected with Ad-Tet alone (fill). Expression of MARCH-VIII was confirmed by surface staining for the known MARCH-VIII substrate TfR. (B): HFFs were transduced with Ad-WT, Ad-K5, Ad-Vpu, or Ad-MARCH-VIII. 24 hours post-transduction cells were harvested and whole cell lysates were analyzed for the abundance of CD44 and CD81 using immunoblot. Samples infected with Ad-MARCH-VIII display significantly reduced levels of both CD44 and CD81 compared to either Mock infected samples or samples infected with other control adenoviruses. Equal protein loading was confirmed by immunoblotting for the ER resident chaperone Bap31 as well as ponceau red staining.

The most likely candidates for novel MARCH-VIII substrates were CD44 and CD81 since both proteins were present at reduced amounts in all three biological replicas. To independently confirm that CD44 and CD81 were down-regulated by MARCH-VIII, HFFs were transduced with either Ad-Tet or Ad-MARCH-VIII together with Ad-Tet and expression of CD44 and CD81 was examined by flow cytometry at 24 h post infection (**Fig. 4A**). Samples transduced with Ad-MARCH-VIII (line) displayed a reduction of both CD44 and CD81 at the cell surface compared to cells infected with Ad-WT (fill). Downregulation of TfR is shown as a control.

To compare the steady state levels of CD44 and CD81 in the presence or absence of MARCH-VIII we used immunoblotting. HFFs were transduced as above and whole cell lysates were analyzed via immunoblot using specific antibodies (**Fig. 4B**). In samples transduced with Ad-MARCH-VIII, a significant reduction of both CD44 and CD81 was seen. In contrast, transduction with adenovirus expressing either KSHV-K5 or HIV-1-Vpu had no effect on either CD44 or CD81 levels. Equal protein loading was confirmed by immunoblotting for Bap31 since total levels of Bap31 do not change upon MARCH expression as discussed above. Thus, it seems that both CD44 and CD81 are specifically affected by MARCH-VIII but not by viral MARCH proteins or unrelated ubiquitin ligases.

### Specificity of CD44 and CD81 downregulation by the MARCH Family

**Figure 5:**
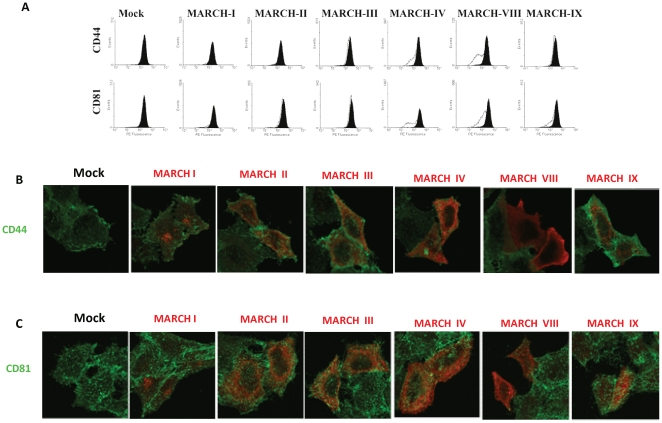
Downregulation of CD44 and CD81 by other members of the MARCH-family. HeLa cells were transfected with plasmids expressing cDNAs for each MARCH protein and the surface expression of CD81 and CD44 were assayed by flow cytometry (A) or confocal immune fluorescence analysis (B and C).

MARCH proteins display specific, but overlapping substrate specificity [13]. Therefore, we tested whether expression of any other member of the MARCH-family would down-regulate CD44 or CD81. For these experiments we used HeLa cells since they can be easily transfected with expression plasmids for each MARCH protein. A plasmid expressing GFP was co-transfected to track transfected cells. 24 hours post transfection, cells were harvested and surface expression of CD44 and CD81 was analyzed using flow cytometry (**Fig. 5A**). Shown are the mean florescence intensities of the GFP+ cells (white line) and GFP- cells (black line) from the same sample. A significant down-regulation of CD81 was observed in cells expressing MARCH-IV which also reduced CD44 expression, albeit not very strongly. This results was highly surprising since the closest homologue of MARCH-VIII is not MARCH-IV, but MARCH-I which demonstrated almost identical substrate specificity to MARCH-VIII in previous experiments by us and others [13], [18], [19].

To determine whether reduced surface levels of CD81 and CD44 were due to degradation or redistribution of these proteins by MARCH-VIII and MARCH-IV we performed immune-fluorescence analysis (IFA). HeLa cells were transfected with each of the Flag-tagged MARCH proteins and co-stained with antibodies for Flag and CD44 (**Fig. 5B**) or CD81 (**Fig. 5C**). Both CD44 and CD81 were absent from cells transfected with MARCH-VIII (in red). In contrast, neighboring cells that were negative for MARCH-VIII were brightly labeled with CD44 or CD81 antibodies (in green). CD81 was also absent from cells transfected with MARCH-IV whereas CD44 was unaffected suggesting that MARCH-IV has only a minimal impact on CD44. Consistent with the flow-cytometry results, all other MARCH proteins did not affect expression levels of CD44 and CD81. These data suggest that CD81 is degraded by both MARCH-IV and MARCH-VIII whereas CD44 is degraded by MARCH-VIII.

### Lysosomal targeting of CD44 and CD81

**Figure 6:**
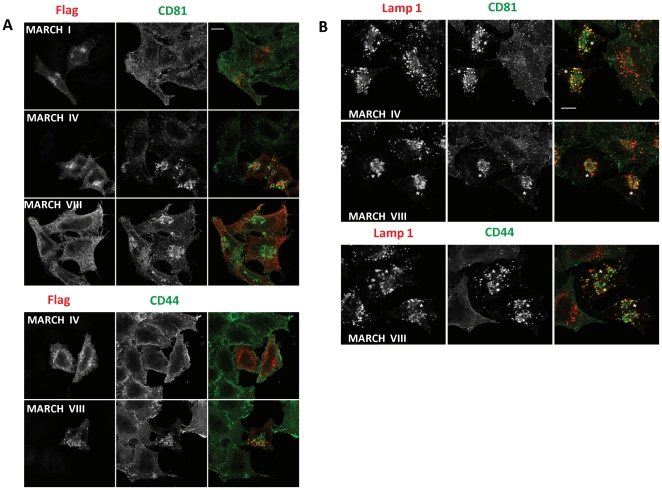
CD81 and CD44 are targeted to lysosomes by MARCH proteins. Flag-tagged MARCH-constructs were transfected into HeLa cells and the cells were treated with 25 mM NH_4_Cl for 2 h. Cells were fixed and stained with anti-Flag and anti-CD81 or anti-CD44 antibodies (A) or stained with either CD81 or anti-CD44 antibodies along with rabbit anti-Lamp1 (B) as described in Materials and Methods. Mouse antibodies were detected with anti-mouse Alexa 488 (green) and rabbit antibodies with anti-rabbit Alexa 594 (red). In B, MARCH-expressing cells, inferred by the altered distribution of CD81 and CD44, are indicated with an asterisk (*). Bar, 10 µm (B).

Most proteins ubiquitinated by MARCH proteins are internalized and targeted for lysosomal degradation via the multi-vesicular body pathway [18]. To determine whether CD81 and CD44 were degraded in lysosomes we performed IFA in the presence of NH_4_Cl which inhibits endosomal maturation. As shown in **Fig. 6A**, CD81 was found in distinct punctate structures upon NH4Cl-treatment in the presence of MARCH-IV or MARCH-VIII whereas CD81 remained at the cell surface in MARCH-I-transfectants. A similar distribution was observed for CD44 in the presence of MARCH-VIII, but not MARCH-IV. To determine whether the vesicles containing CD81 and CD44 in the presence of MARCH proteins represented late endosomes or lysosomes we co-stained CD81 and CD44 in MARCH-transfected cells with the lysosomal marker Lamp-1. In cells expressing MARCH-VIII and MARCH-IV (**Fig. 6B**, labeled with *), CD81 (in green) partially co-localized with Lamp-1 (in red) whereas no co-localization was observed in neighboring untransfected cells. A similar co-staining was observed for CD44 upon transfection of MARCH-VIII (**Fig. 6B**). Therefore, we conclude that CD81 is targeted for lysosomal destruction by MARCH-IV and MARCH-VIII whereas CD44 is directed to lysosoaml degradation only by MARCH-VIII.

### Knockdown of MARCH-IV results in upregulation of CD81

**Figure 7:**
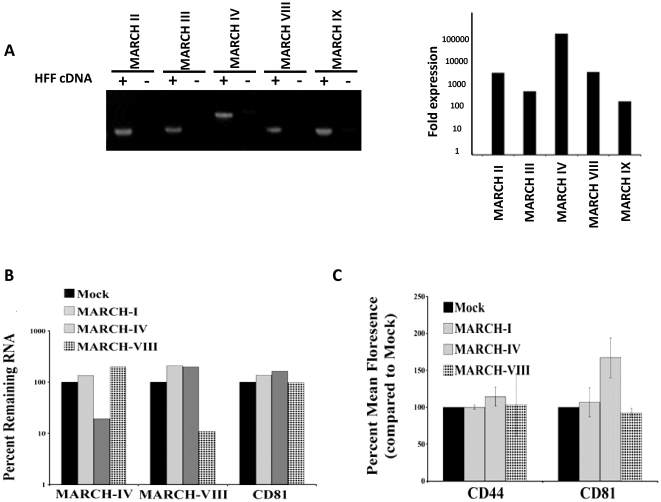
Depletion of MARCH-IV affects surface expression of CD81. (A): MARCH expression in HFF was analyzed using real-time PCR and the PCR products were separated on a 1% agarose gel and visualized using sybr green (left panel). MARCH mRNA expression is shown as the fold change between samples containing HFF cDNA and no template control samples (right panel). (B): HFFs were treated with siRNA against MARCH-I, -IV, or –VIII as indicated. Cells were treated with siRNA four times over the course of 7 days. The success of each siRNA treatment was determined by monitoring the reduction of MARCH mRNA levels using real time quantitative PCR. (C): In parallel, cells were harvested via trypsinization and the surface levels of CD44 and CD81 were measured using flow cytometry. Graphs are displayed as percent mean florescence intensity.

Since HHFs express detectable levels of endogenous MARCH-VIII and MARCH-IV **(** **Fig. 7A**), we wondered whether the steady state level of CD44 or CD81 was affected by the constitutive, albeit low level expression of these ubiquitin ligases. Therefore, HFFs were treated with siRNA against both MARCH proteins either separately or in combination. Knockdown of each MARCH protein was confirmed by real time PCR (**Fig. 7B**). Treatment with either MARCH-IV or MARCH-VIII siRNA reduced corresponding MARCH RNA levels by around 90%. RNA levels for MARCH-IV or MARCH-VIII were unchanged by treatment with the mismatched siRNA, showing that depletion was sequence specific. Additionally, treatment with a siRNA against MARCH-I had no effect on RNA levels for either MARCH-IV or MARCH-VIII. Since MARCH-I is not expressed in HFF cells (data not shown) this served as a control. Following depletion of MARCH-IV or MARCH-VIII, cells were harvested and the surface levels of CD44 and CD81 were measured using flow-cytometry. Treatment with siRNA against either MARCH-IV or MARCH-VIII showed no effect on the surface levels of CD44 (**Fig. 7C**). Similarly, CD81 levels did not change upon MARCH-VIII knockdown. In contrast, treatment with siRNA against MARCH-IV resulted in a 50% increase of CD81 surface levels compared to either untreated cells or cells treated with other siRNA (**Fig. 7C**). While modest, this increase was reproducible over five separate experiments and statistically significant with a *p* value<0.01 compared to treatment with siRNA against MARCH-VII. This increase was not due to increased transcription of CD81 since treatment with MARCH-siRNAs did not increase levels of CD81 mRNA (**Fig. 7B**). Co-transfection of siRNA against MARCH-IV and MARCH-VIII did not further increase CD81 levels suggesting that endogenous MARCH-VIII might be expressed at levels that are too low to influence CD81 expression whereas MARCH-IV is more highly expressed as indicated by qPCR (**Fig. 7A**). However, endogenous expression levels seem to be too low for both MARCH proteins to influence CD44 turnover. It remains to be demonstrated whether CD44 and CD81 are regulated by MARCH-VIII in cells expressing higher levels of MARCH-VIII such as immature dendritic cells. However, these data clearly show that endogenous MARCH-IV constitutively affects CD81 levels.

## Discussion

The goal of this study was to use an unbiased proteomics approach to identify cell surface proteins regulated by transmembrane ubiquitin ligases of the MARCH protein family. Using the viral MARCH homologue KSHV-K5, we had previously demonstrated that it is possible to identify novel targets for both the protein used in the proteomics study, as well as other members of this family [14]. For instance, ALCAM and BST2/Tetherin were identified as novel substrates for KSHV-K5. Follow up experiments, however, demonstrated that ALCAM was also down-regulated by MARCH-VIII and Myxomavirus M153R whereas BST2/Tetherin was also down-regulated by MARCH-VIII and HIV-1 Vpu. Similarly, we now show that CD81 was also down-regulated by MARCH-IV. Although a modest downregulation of CD44 was observed with MARCH-IV by flow cytometry, this was not observed by IFA. Interestingly, expression of CD44 or CD81 was not affected by the viral MARCH proteins tested, suggesting that these endogenous substrates are specifically targeted cellular MARCH proteins. Also surprising was the observation that MARCH-I and MARCH-IX, which are closely related to MARCH-VIII and MARCH-IV, respectively, did not affect either CD81 or CD44. This is in contrast to our previous observations which showed that sequence homology strongly correlated with substrate specificity. Why a similar correlation is not observed for CD81 and CD44 is presently not known.

While both CD44 and CD81 are widely expressed on different cell types, their regulation has been shown to play a particularly important role in the developing immune system. Like most targets of MARCH proteins, CD44 is a type I transmembrane glycoprotein. CD44 occurs in many splice variants and connects a variety of extracellular matrix proteins, most notably hyaluronic acid (HA), to the cell surface [33]. CD44 plays a crucial role in inflammation [49] and in cancer progression [50]. It is also involved in leukocyte recruitment [51] and regulates T cell interactions during T cell development [52]. Since CD44 exists in many isoforms and is extensively spliced [53], MARCH-VIII dependent down-regulation might be variant-specific. However, this possibility has yet to be investigated. Although elimination of MARCH-VIII from fibroblasts did not change the steady state levels of CD44, it is likely that CD44 is affected in more dynamic situations where MARCH proteins are regulated, such as during dendritic cell maturation [54]. MARCH- VIII could also play a role for the HA-induced endocytosis of CD44 and its subsequent targeting to the multi-vesicular bodies (MVB) [55] since most MARCH-VIII targets reach the MVB [13], [16].

In contrast to CD44, CD81 surface levels were increased upon depletion of MARCH-IV, suggesting that MARCH-IV is involved in the natural turnover of CD81 in fibroblasts. While depletion of MARCH-VIII had no effect on CD81 levels in fibroblasts, it is possible that higher MARCH-VIII levels in other cell types or tissues might affect CD81. Unlike most MARCH-targets, which are single-span transmembrane proteins, CD81 belongs to the tetraspanin family, four-transmembrane proteins that form the tetraspanin-web as a membrane microdomain which laterally incorporates multiple transmembrane proteins [56]. Since several of the CD81-interacting proteins have been shown to be down-regulated by MARCH-IV or MARCH-VIII it is possible that CD81 surface expression is indirectly affected via these *bona-fide* targets. Alternatively, it is conceivable that some of these proteins could be down-regulated as a consequence of CD81 ubiquitination and degradation. In fact, targeting a protein with widespread protein:protein interactions, such as CD81, might explain how MARCH proteins down-regulate such a variety of substrates, with no sequence similarity. Since our observations that MARCH-IV lacking a functional RING-CH domain and mutants of MARCH-VIII which are unable to down-regulate B7.2 both fail to down-regulate CD81 are consistent with either model, further work is required to distinguish between these possibilities. The validation of both CD81 and CD44 by independent methods confirms our previous conclusion that three biological replica experiments give a high level of confidence for the specificity of the results [14]. Since ALCAM, a known substrate of MARCH-VIII, was identified in only two of the three experiments, however, we selected two additional potential candidate proteins, CD9 and Bap31, for validation based on their sub-cellular localization as well as their known interaction with MARCH-VIII substrates. Only Bap31, however, confirmed using independent methods, indicating that the false-positive rate is high in these types of experiments when biological replicas are limited. This is primarily due to the inaccuracy of quantitation using the mass spectroscope.

Unlike CD44 and CD81, steady state levels of Bap31 were not diminished following expression of MARCH-VIII. Instead, we found that MARCH-VIII bound to Bap31 and prevented Bap31 from reaching the cell surface. We further observed that Bap31 interacts with most MARCH proteins, suggesting that Bap31 chaperones the folding, assembly or intracellular transport of this protein family. Interestingly, depletion of Bap31 resulted in lower amounts of MARCH-VIII expressed on the cell surface. Since Bap31 is known to function as a forward cargo molecule for several other proteins [37], [39], [42], [44] we conclude that that Bap31 is involved in sub-cellular sorting of MARCH proteins. Interestingly, the MARCH-family and their viral homologues have been shown to interact with several other intracellular trafficking molecules. Depletion of PACS2 inhibited K5 mediated degradation of newly synthesized CD31, suggesting that it played a key role in K5 function [12]. Several members of the MARCH-family have been shown to interact directly with members of the syntaxin family of SNARE proteins [14], [57], [58]. However, the functional consequences of these interactions are not currently understood.

Truncated versions of MARCH-VIII that do not interact with syntaxins are still able to interact with Bap31 [14] suggesting that the interaction with Bap31 occurs in the transmembrane region. This is consistent with previous findings that Bap31's interaction with MHC I, IgD, cellubrevin, and p450 localizes to the transmembrane domains [40], [42], [44]. Interestingly, expression of Bap31 is required for surface expression of CD81 [36]. Thus, inhibition of Bap31 might play some role in MARCH-IV and –VIII down-regulation of CD81.

While depletion of Bap31 affected intracellular transport of MARCH-VIII, it did not prevent MARCH-VIII-mediated down-regulation of transfected CD86 (B7.2) (data not shown). This result, however, is most likely explained by insufficient knockdown of Bap31, resulting in sufficient amounts of MARCH-VIII available at the cell surface to ubiquitinate its substrates.

In summary, we present a systematic identification of endogenous substrates for the MARCH gene family. By using this non-biased approach to substrate discovery, we further demonstrate that the tetraspanin CD81 is down-regulated by both MARCH-IV and –VIII thus expanding the structural range of possible MARCH substrates. We were also able to show that depletion of endogenous MARCH-IV increased the surface expression of CD81, the first time that a substrate of MARCH-IV has been affected by depletion of endogenous protein. Thus, these approaches will be useful in unraveling the function of this class of proteins.
